# The interplay between HPIP and casein kinase 1α promotes renal cell carcinoma growth and metastasis via activation of mTOR pathway

**DOI:** 10.1038/oncsis.2016.44

**Published:** 2016-10-03

**Authors:** H Mai, X Xu, G Mei, T Hong, J Huang, T Wang, Z Yan, Y Li, Y Liang, L Li, S Jin, W You, Y Ma, L Chen, Q Ye

**Affiliations:** 1 Department of Medical Molecular Biology, Beijing Institute of Biotechnology, Beijing, People's Republic of China; 2 Department of Urology, Affiliated Hospital of Academy of Military Medical Sciences, Beijing, People's Republic of China; 3 Department of Oncology, Affiliated Hospital of Academy of Military Medical Sciences, Beijing, People's Republic of China; 4 Department of Gynecology and Obstetrics, PLA General Hospital, Beijing, People's Republic of China; 5 Department of Oncology, PLA General Hospital, Beijing, People's Republic of China; 6 Department of Thoracic Surgery, PLA General Hospital, Beijing, People's Republic of China

## Abstract

Hematopoietic pre-B cell leukemia transcription factor (PBX)-interacting protein (HPIP) was shown to be crucial during the development and progression of a variety of tumors. However, the role of HPIP in renal cell carcinoma (RCC) is unknown. Here we report that HPIP is upregulated in most RCC patients, positively correlates with tumor size, high Fuhrman grade and preoperative metastasis, and predicts poor clinical outcomes. Mechanistically, we identified casein kinase 1α (CK1α), a critical regulator of tumorigenesis and metastasis, as a novel HPIP-interacting protein. HPIP facilitates RCC cell growth, migration, invasion and epithelial–mesenchymal transition depending on its interaction with CK1α. Activation of mammalian target of rapamycin pathways by HPIP is partly dependent on CK1α and is required for HPIP modulation of RCC cell proliferation and migration. HPIP knockdown suppresses renal tumor growth and metastasis in nude mice through CK1α. Moreover, expression of CK1α is positively correlated with HPIP in RCC samples, and also predicts poor clinical outcome-like expression of HPIP. Taken together, our data demonstrate the critical regulatory role of the HPIP–CK1α interaction in RCC, and suggest that HPIP and CK1α may be potential targets for RCC therapy.

## Introduction

Renal cell carcinoma (RCC) is the most common type of kidney cancer in adults, responsible for ~90–95% of kidney malignancies. Surgical operation remains the most effective treatment for RCC, but up to 30% newly diagnosed patients develop metastasis, with the 5-year survival rate of <10%, and 20–30% post-surgery treatment cases eventually develop recurrence.^1^ As RCC is resistant to traditional chemotherapy, hormonal therapy or radiation therapy, further investigation of the molecular mechanisms underlying RCC tumorigenesis and progression is crucial for individual treatment of RCC.

Hematopoietic pre-B-cell leukemia transcription factor (PBX)-interacting protein (HPIP), a co-repressor for pre-B-cell leukemia homeobox 1 (PBX1), is known to act as a promoter during tumorigenesis. We and others have reported that HPIP is upregulated in varieties of cancers, such as breast infiltrative ductal carcinoma,^2, 3^ astrocytoma,^4^ liver cancer,^5, 6^ oral cell carcinoma,^7^ colorectal cancer^8^ and so on. However, the role of HPIP in RCC remains unknown.

In the current study, we first investigated the role of HPIP in RCC growth and metastasis both in *vitro* and in *vivo*. More importantly, we identified casein kinase 1α (CK1α), a critical regulator of tumorigenesis and metastasis, as a novel HPIP-interacting protein, thus linking the oncogenic ability of HPIP to its interaction with CK1α. In addition, we found that HPIP expression positively correlates with CK1α, and the expression of both proteins predicts poorer outcome in RCC patients, demonstrating the critical clinical significance of the HPIP–CK1α interaction in RCC.

## Results

### Clinical significance of HPIP expression in human RCC samples

**Figure 1:**
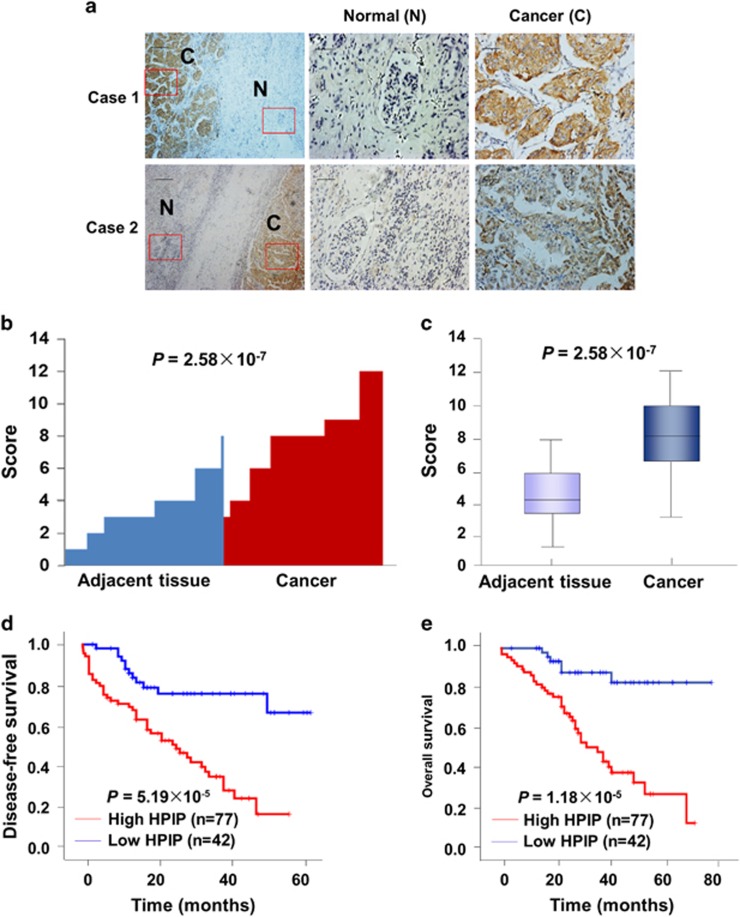
HPIP expression is upregulated in RCC patients and predicts poorer outcome. (**a**) Representative immunohistochemical staining of HPIP protein in renal cancer tissue (middle) and matched adjacent normal renal tissue (right). The boxed areas in the left images are magnified in the middle and right images. Scale bar, 200 μm (left), 50 μm (middle and right). (**b**, **c**) HPIP expression scores were displayed in bar chart (**b**) and box-and-whisker plots (**c**), and compared using Mann–Whitney *U*-test. (**d**, **e**) Kaplan–Meier estimates of disease-free survival (**d**) and overall survival (**e**) of RCC patients. Marks on graph lines represent censored samples.

To investigate the clinical significance of HPIP in RCC, first, we assessed HPIP expression by immunohistochemical staining of tissues consisting of 119 pairs of human RCC tumors and their matched non-tumor renal tissues. HPIP was distributed mainly in the cytoplasm. On the basis of HPIP scores, HPIP expression was significantly upregulated in RCC patients (*P*=2.58 × 10^−7^; Figures 1a–c). To further investigate the relevance between HPIP and clinicopathological characteristics, we divided the RCC samples into two groups according to their HPIP expression levels. As expected, the high HPIP expression group showed higher incidences of larger tumor sizes (*P*=2.550 × 10^−4^), higher Fuhrman grade (*P*=8.759 × 10^−5^) and preoperative metastasis (*P*=0.005; Table 1). More importantly, Kaplan–Meier survival analysis of HPIP expression revealed that the patients with high HPIP scores had shorter disease-free survival and overall survival than those with low expression of HPIP (disease-free survival: *P*=5.19 × 10^−5^ overall survival: *P*=1.18 × 10^−5^; Figures 1d and e), indicating that HPIP predicts poorer clinical outcome of RCC. The specificity of anti-HPIP antibody was confirmed by immunohistochemical staining of RCC tissues incubated with anti-HPIP pre-incubated with its antigen (Supplementary Figure 1A) and immunoblotting of lysates from Caki-1 and 786-O RCC cells infected with HPIP short hairpin RNA (shRNA; Supplementary Figure 1B). Taken together, these data strongly suggest an important pathological role of HPIP in RCC.

### Identification of CK1α as a novel HPIP-interacting protein

**Figure 2:**
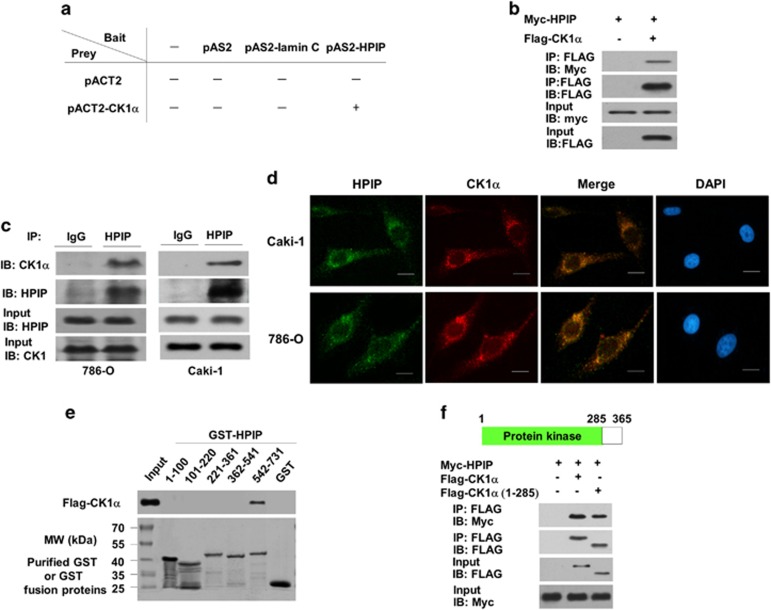
Identification of CK1α as a novel HPIP-interacting protein. (**a**) Yeast CG1945 cells were transformed with the indicated plasmids (bait and prey for the two-hybrid assay) and grown on selective media. Positive interaction is indicative of colonies that grow on selective media and have β-galactosidase activity. (**b**) 293T cells were co-transfected with expression plasmids as indicated. Immunoprecipitation (IP) was performed using anti-FLAG monoclonal antibody, and immunoblotted (IB) with anti-myc antibody. (**c**) 786-O cells or Caki-1 cells were immunoprecipitated (IP) with the indicated antibodies or preimmune control serum (IgG). Precipitates were analyzed by immunoblot using the indicated antibodies. (**d**) HPIP colocalizes with CK1α. 786-O cells or Caki-1 cells were stained with anti-HPIP and anti-CK1α before visualization by confocal microscopy. The nuclei were stained with DAPI (blue). (**e**) Mapping of the HPIP region responsible for interaction with CK1α. GST pull-down was performed with various HPIP deletion mutants. (**f**) Mapping of the CK1α region responsible for interaction with HPIP. GST pull-down was performed as in **e**.

To investigate the possible mechanisms of how HPIP regulates RCC, we screened proteins that could interact with HPIP using yeast two-hybrid system. CK1α, a key regulator of tumorigenesis and metastasis,^9^ was identified as one of the HPIP-interacting proteins. The specificity of this interaction was confirmed in a direct two-hybrid binding assay (Figure 2a). To determine whether CK1α specifically interacts with HPIP *in vivo*, 293 T cells were co-transfected with FLAG-CK1α and Myc-HPIP, and immunoprecipitated (IP) from cell lysates by anti-FLAG and analyzed for HPIP binding by immunoblotting (IB). FLAG-CK1α could be co-immunoprecipitated in the presence of Myc-HPIP (Figure 2b). Endogenous CK1α was also found to be specifically interacted with endogenous HPIP in both 786-O and Caki-1 cells by Co-IP (Figure 2c). Immunofluorescence assay further confirmed the colocalization of the two proteins (Figure 2d). These data strongly suggest that CK1α specifically interacts with HPIP in *vivo*.

To define which regions of HPIP bind to CK1α, GST pull-down experiments were performed. CK1α only bound HPIP (542–731), but not other HPIP fragments tested (Figure 2e). These results indicate that the C-terminal region of HPIP is critical for its interaction with CK1α.

To delineate the domains in the CK1α that mediate the protein–protein interaction with HPIP, Co-IP experiments were performed. Series of mutant FLAG-CK1α fusion proteins were used in IP experiments. As shown in Figure 2f, HPIP interacted with full-length CK1α and CK1α (1–285) containing the kinase domain. These results suggest that the CK1α kinase domain is critical for its interaction with HPIP.

### Interaction of HPIP and CK1α is required for HPIP modulation of RCC proliferation

**Figure 3:**
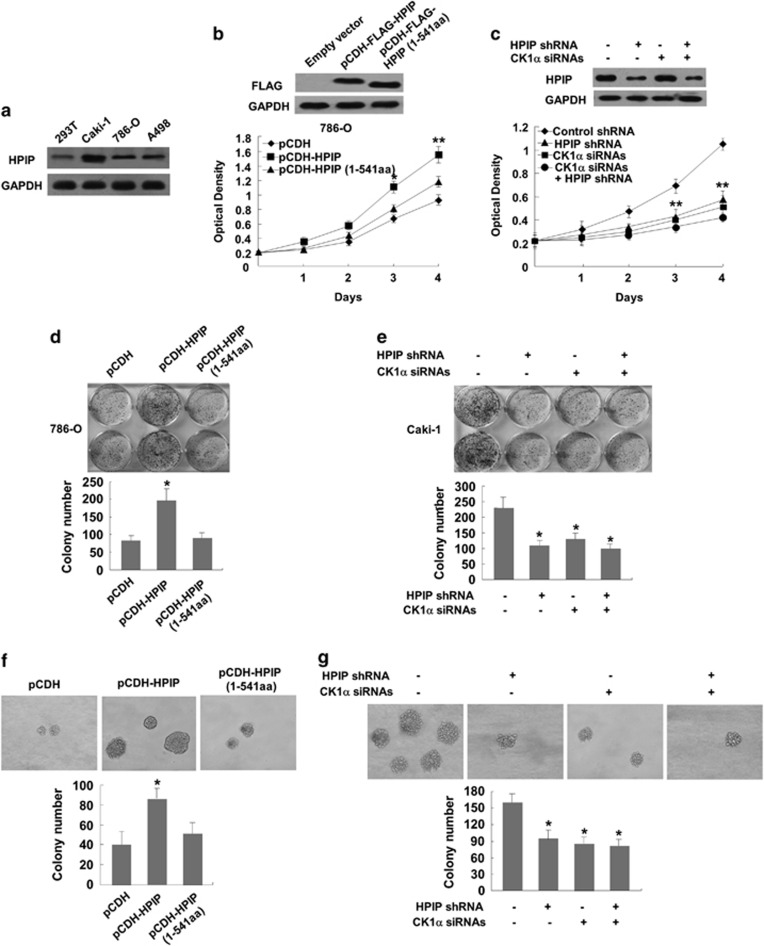
Interaction of HPIP with CK1α is required for HPIP modulation of RCC proliferation. (**a**) Total proteins extracted from the indicated RCC cell lines were analyzed by immunoblotting with anti-HPIP. GAPDH was used as a loading control. (**b**) 786-O cells infected with Flag-tagged HPIP, Flag-tagged HPIP (1-541) or empty vector were grown in regular medium and harvested at the indicated times. Cell number was determined by CCK-8 assay. The representative immunoblot with FLAG-HRP indicates HPIP expression levels. (**c**) Caki-1 cells stably infected with HPIP shRNA and control shRNA were transfected with CK1α siRNAs and analyzed as in **b**. (**d**, **e**) Colony formation assays using 786-O cells (**d**) and Caki-1 cells (**e**). Histograms show the colony number. (**f**, **g**) RCC cells were plated in soft agar and assayed for the colony number and comparison of colony diameters. Representative images show colonies in soft agar (left panels). All values shown are the mean±s.d. of triplicate measurements. Experiments were repeated three times with similar results. All values shown are mean±s.d. of triplicate measurements and have been repeated three times with similar results (*P<0.05 versus empty vector or control shRNA, **P<0.01 versus empty vector or control shRNA).

Next, the effect of HPIP overexpression or knockdown of endogenous HPIP protein on RCC growth was investigated. All three RCC cell lines (Caki-1, 786-O and A498) and one human embryonic kidney cell line (293T) expressed endogenous HPIP protein (Figure 3a). Among them, Caki-1 cell line expressed HPIP at the highest level, 293T cell line expressed HPIP at the lowest level, and 786-O and A498 cell line expressed HPIP at the medium level. Therefore, we chose Caki-1 to knockdown HPIP and 786-O to overexpress HPIP. 786-O cells with HPIP overexpression grew much faster than those infected with empty vector. Importantly, HPIP (1-541) that lacks CK1α-binding site abolished the ability of HPIP to promote RCC growth (Figure 3b). In contrast, Caki-1 cells infected with HPIP shRNA grew more slowly than those infected with control shRNA. However, when CK1α was knocked down, HPIP failed to regulate RCC proliferation (Figure 3c). Colony formation and soft agar assays revealed similar trends to that of growth curves mentioned above (Figures 3d–g). These data collectively suggest that interaction between HPIP and CK1α is required for HPIP promotion of RCC proliferation.

### HPIP promotes CK1α-dependent RCC cell migration and invasion with increased EMT

**Figure 4:**
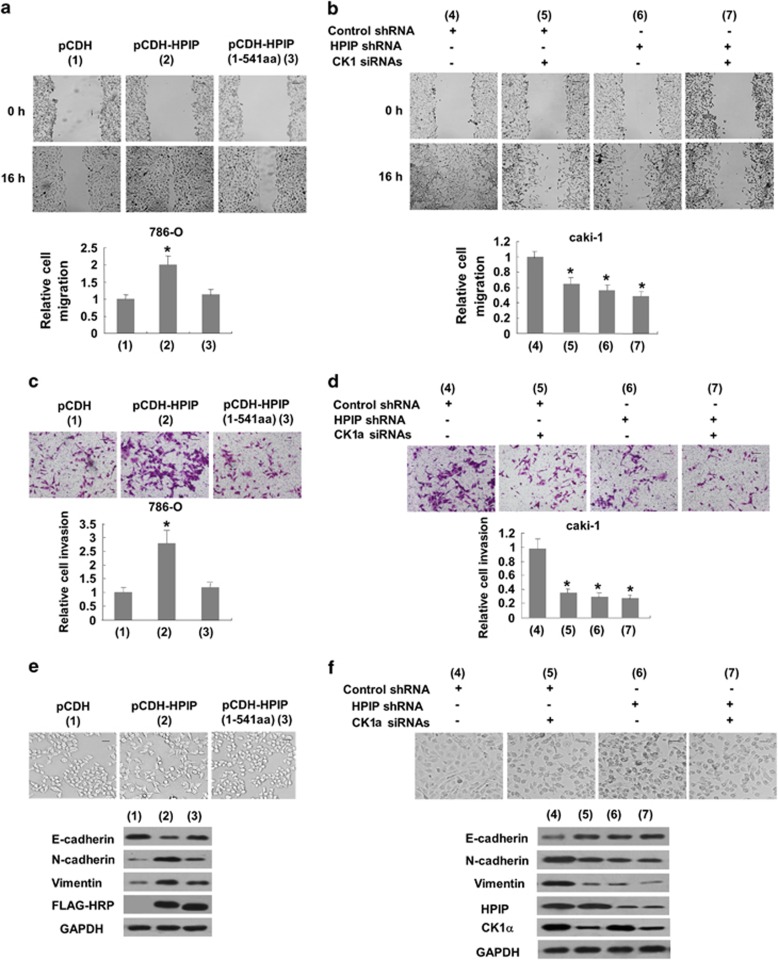
HPIP promotes CK1α-dependent RCC cell migration and invasion with increased EMT. (**a**) Migration of 786-O cells infected with Flag-tagged HPIP, Flag-tagged HPIP (1-541) or empty vector. (**b**) Caki-1 cells stably infected with HPIP shRNA or control shRNA were transfected with CK1α siRNAs and cell migration was determined by wound healing assay. The experiments have been repeated three times with similar trends and the image displayed is one of the representative results. (**c**) Cell invasion of 786-O cells infected as in **a**. (**d**) Caki-1 cells infected and transfected as in **b** were assessed by the Matrigel invasion chamber. Invasive cells were fixed and stained with crystal violet. Scale bar, 100 μm. The number of invaded cells in (**c**) 786-O or (**d**) Caki-1 cells was counted. All values shown are mean±s.d. of triplicate measurements and have been repeated three times with similar results. *P<0.05 versus empty vector or control shRNA. (**e**, **f**) Representative western blot analysis of 786-O cells infected as in **a** and Caki-1 cells infected and transfected as in **b**. Morphologic changes are shown in the photographs. Scale bar, 100 μm.

**Figure 5:**
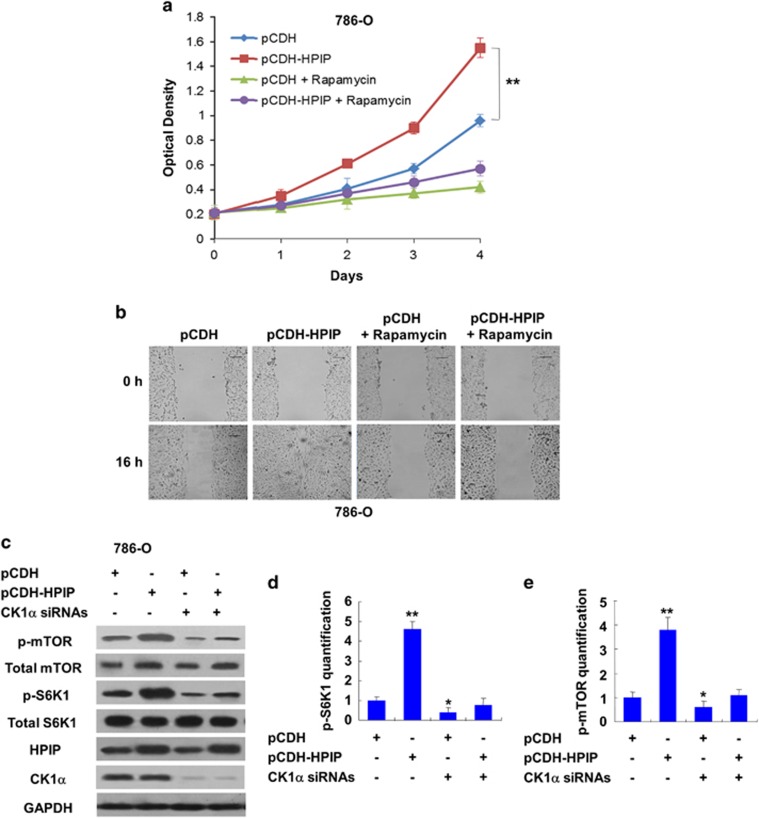
HPIP increases RCC cell proliferation and migration partly dependent on CK1α-mediated activation of mTOR pathway. (**a**) 786-O cells were infected with lentivirus expressing HPIP (pCDH-HPIP) or empty vector (pCDH), and were treated for 24 h with 10 μm Rapamycin. After 24 h, the culture medium was changed with fresh drug-free medium, and the cells were grown for the indicated times. Cell number was determined by CCK-8 assay. (**b**) Wound healing assays for 786-O cells infected as in **a** and treated with Rapamycin for 24 h. Scale bar, 100 μm. (**c**) Representative western blot analysis of 786-O cells stably infected with pCDH-HPIP or pCDH empty vector and transfected with CK1α siRNAs. All values shown are mean±s.d. of triplicate measurements and have been repeated three times with similar results (*P<0.05, **P<0.01). (**d**, **e**) Quantification analysis of p-mTOR (**d**) and p-S6K1 (**e**) density normalized to GAPDH in western blot result displayed in (**c**).

To test the effects of HPIP on RCC cell migration and invasion, wound-healing and transwell invasion assays were used. Wound-healing assay demonstrated that overexpression of HPIP in 786-O cells increased migration ability (Figure 4a), while knockdown of HPIP in Caki-1 cells reduced cell motility (Figure 4b). Transwell invasion assay revealed that overexpression of HPIP in 786-O cells enhanced the number of invaded cells (Figure 4c), whereas knockdown of HPIP in Caki-1 cells reduced the number of invaded cells (Figure 5d). Moreover, HPIP mutant (1-541) failed to promote RCC migration and invasion, and knockdown of CK1α greatly impaired the effect of HPIP on cell migration and invasion (Figures 4a–d).

Consistent with the results of HPIP modulation of RCC cell migration and invasion, overexpression of HPIP promoted EMT, which has been shown to have a critical role in cancer cell migration and invasion,^10^ with morphologic changes from a polarized epithelial phenotype to an elongated fibroblastoid phenotype and with the decreased expression of the epithelial marker E-cadherin and increased expression of N-cadherin and Vimentin, two mesenchymal markers. HPIP mutant (1-541) failed to regulate EMT (Figure 4e). On the other hand, knockdown of HPIP repressed EMT, which was impaired when CK1αwas knocked down (Figure 4f). These data indicate that CK1α is responsible for HPIP promotion of RCC cell migration and invasion with increased EMT.

### HPIP increases RCC cell proliferation and migration partially through CK1α-mediated activation of mTOR pathway

Mammalian Target of Rapamycin (mTOR) kinase, which regulates cell growth, survival, invasion and metastasis, is a critical player in tumorigenesis and progression.^11^ As HPIP has been shown to activate mTOR signaling in liver cancer cells,^6^ we investigated whether activation of mTOR signaling is responsible for HPIP modulation of RCC cell proliferation and migration. We treated HPIP-overexpressing 786-O cells with Rapamycin, which is mTOR inhibitor.^12^ As expected, HPIP overexpression in 786-O cells promoted cell proliferation and migration. Importantly, treatment with Rapamycin greatly alleviated the ability of HPIP to regulate RCC cell proliferation and migration (Figures 5a and b). These results suggest that activation of mTOR is responsible for HPIP modulation of RCC cell proliferation and migration.

CK1α was reported to activate mTOR signaling by degradation of the mTOR kinase inhibitor DEPTOR.^13^ To further investigate the relationship between mTOR activation by HPIP and CK1α status, we knocked down CK1α and tested the effect of HPIP on mTOR signaling. Quantification data of p-mTOR and p-S6K1 in western blot analysis demonstrated that the ability of HPIP to activate mTOR signaling was impaired when CK1α was depleted, which indicates that HPIP activates mTOR pathway partly dependent on CK1α (Figures 5c–e).

### HPIP modulates RCC cell growth and metastasis in nude mice via CK1α

**Figure 6:**
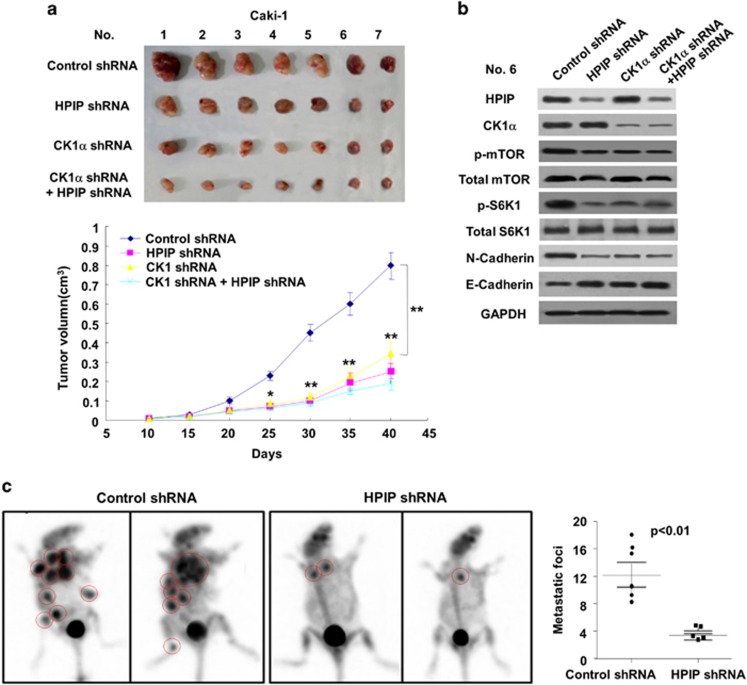
HPIP modulates RCC cell growth through CK1α in nude mice. (**a**) Caki-1 cells stably infected with lentiviruses carrying the indicated constructs were injected into nude mice. At the indicated times, tumors were measured with Vernier calipers (mean±s.d.; *n*=7). **P<0.01 versus corresponding control shRNA. (**b**) Immunoblot analysis of representative excised tumor from **a**. (**c**) Small-PET imaging of RCC cell metastasis in nude mice (*n*=6) at 50 days after intravenous injection of control shRNA- or HPIP shRNA-infected Caki-1 cells via the lateral tail vein. Images and radioactivity showed that HPIP knockdown clearly repressed the number of the pulmonary and bone spread nodules. Red circles indicate tumor foci. The two-tailed Student's *t*-test was used to compare the metastatic numbers in the two groups (**P<0.01).

Next, we tested the effects of HPIP and CK1α on cancer cell proliferation in a xenograft mouse model. Consistent with the results of the cell proliferation assays, mice inoculated with HPIP or CK1α knockdown Caki-1 cells grew more slowly than those cells stably infected with control shRNA and parental cells (Figure 6a). Importantly, CK1α knockdown abrogated the ability of HPIP knockdown to repress Caki-1 tumor growth. As expected, the Caki-1 tumors in mice inoculated with HPIP shRNA or CK1α shRNA showed decreased expression of HPIP/CK1α, and N-cadherin, and increased expression of E-cadherin. In addition, CK1α knockdown abolished the ability of HPIP to regulate the expression of the proteins mentioned above (Figure 6b). Moreover, we used Caki-1, a metastatic RCC cell line derived from a human metastatic clear cell renal carcinoma, to measure the effect of HPIP knockdown on RCC metastasis. Mice injected with HPIP shRNA Caki-1 cells showed a significant decrease in lung and bone metastatic burden compared with the control shRNA group (Figure 6c). These findings strongly support the role of HPIP as a promoter of tumor dissemination.

### Expression of CK1α and the correlation between HPIP and CK1α in human RCC samples

**Figure 7:**
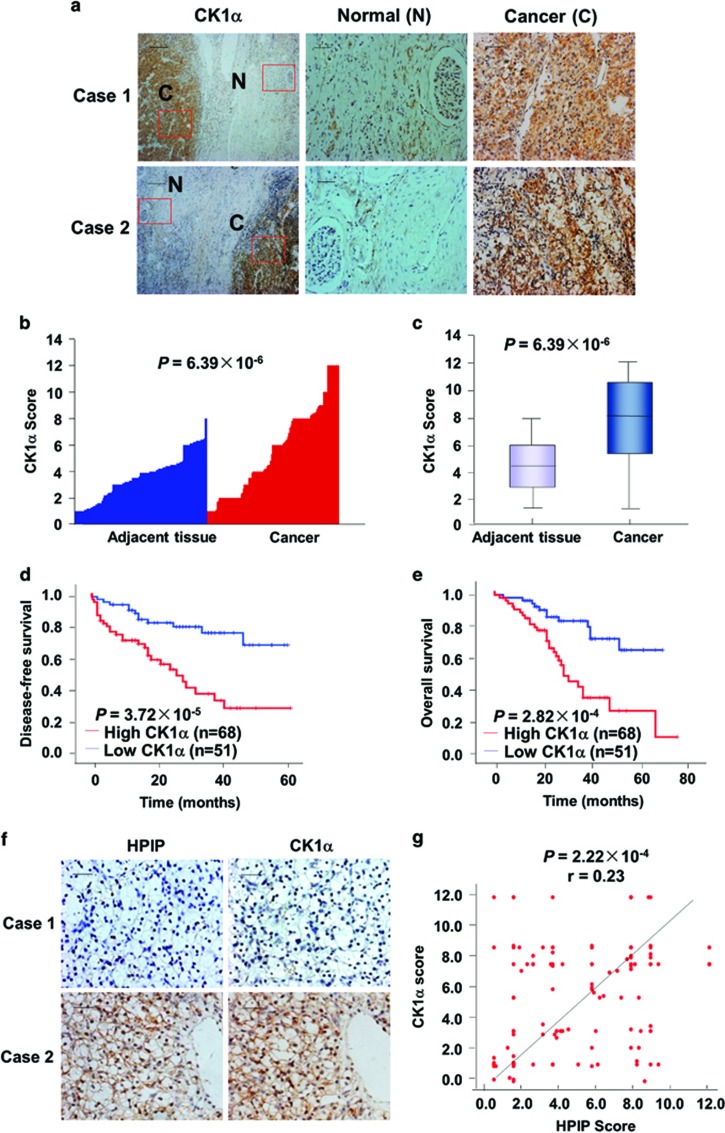
Expression of CK1α and its correlation with HPIP in RCC patients. (**a**) Representative immunohistochemical staining of CK1α in cancerous (**c**) renal tissues and adjacent normal (N) renal tissues. The boxed areas in the left images are magnified in the middle and right images. Scale bars, 250 μm (left) and 50 μm (middle and right). (**b**, **c**) CK1α expression scores were (**b**) plotted and (**c**) compared by the Mann–Whitney *U*-test. (**d**, **e**) Kaplan–Meier survival curves and the log-rank test were used to assess (**d**) disease-free survival and (**e**) overall survival according to the low and high scores of CK1α in RCC patients. (**f**) The relationship between CK1α and HPIP expression was assessed by Spearman rank correlation analysis of RCC samples. Symbols represent individual samples.

Until now, several reports link altered CK1α expression to cancer.^14^ However, the clinical significance of CK1α in RCC remains to be investigated. We assessed CK1α expression by immunohistochemical staining in 119 pairs of human renal carcinomas and their matched adjacent non-tumor renal tissues. On the basis of immunohistochemical staining scores, CK1α expression was significantly upregulated in RCC patients (*P*=6.39 × 10^−6^) (Figures 7a–c). Moreover, Kaplan–Meier survival analysis of CK1α expression revealed that patients with high CK1α scores had poorer disease-free survival (DFS) (*P*=3.72 × 10^−5^) and overall survival (*P*=2.82 × 10^−4^) than those with low CK1α scores, indicating that CK1α predicts poorer clinical outcome (Figures 7d and e). We confirmed the specificity of the anti-CK1α antibody by pre-incubation of the antibody with its antigen before immunohistochemical staining and immunoblotting of lysates from 786-O cells and Caki-1 cells transfected with CK1α shRNA (Supplementary Figures 2A and B). In addition, expression of CK1α was positively correlated with HPIP expression in RCC samples (*P*=2.22 × 10^−4^, *r*=0.23) (Figures 7f and g). Taken together, these data strongly suggest the important pathological roles of HPIP and CK1α in RCC.

## Discussion

Recently, accumulating evidence has revealed that HPIP functions as a promoter of the development and progression of cancers, including breast cancer,^2, 3^ astrocytoma,^4^ liver cancer,^5, 6^ oral cell carcinoma,^7^ colorectal cancer^8^ and so on. However, the role of HPIP in RCC remains unclear. In this study, we report for the first time that HPIP is overexpressed in RCC patients and predicts poor prognosis. Mechanistically, we identify CK1α as a novel HPIP-interacting protein, thus linking the oncogenic ability of HPIP to its interaction with CK1α. We further show that, like HPIP, CK1α is also upregulated in RCC patients and predicts poor clinical outcome. This is the first time to show the clinical significance, especially the prognostic value of CK1α in RCC although altered CK1 isoforms expression have been reported to contribute to tumorigenesis and metastatic behavior in varieties of tumors.^15, 16, 17, 18^ Our data suggest that HPIP and CK1α may be potential targets for RCC therapy.

Exploring the molecular mechanisms of cancer cell growth and metastasis is of critical importance. mTOR signaling that regulates cell growth, proliferation and survival is frequently activated in many tumors including RCC.^19, 20, 21^ Our data suggest that HPIP activates mTOR signaling in RCC cells which is consistent with our previous findings in liver cancer.^6^ In our published work, we demonstrate that HPIP controls mTOR signaling by a cooperative mechanism involving both modulation of mTOR phosphorylation and mTOR expression. mTOR expression has been proved to be regulated by AKT/ERK-FOXO4-ATF5 pathway, however, the detailed mechanism of mTOR phosphorylation regulated by HPIP needs to be further investigated. Besides, the testified mechanism that activation of mTOR phosphorylation by HPIP is caused by activation of protein kinase B (AKT) and extracellular signal-regulated kinase (ERK), in our current study, we also established the necessity of CK1α in the process of mTOR phosphorylation regulated by HPIP. When CK1α is depleted, the ability of HPIP to regulate mTOR phosphorylation and its downstream factors is greatly impaired, indicating the importance of CK1α in contributing to mTOR activation led by HPIP. It should be noted that the activation effect of HPIP on mTOR phosphorylation is just impaired when CK1α is depleted but not abolished, as the regulation of mTOR abundance by HPIP still exists. Thus, we provide new details underlying HPIP modulation of mTOR phosphorylation, at least partly dependent on CK1α. As for how HPIP and CK1α control mTOR signaling, it still needs further investigation.

Invasion and metastasis are major reasons for the poor prognosis of RCC patients. Previous studies have indicated that many molecular mechanisms contribute to the metastasis of RCC, including EMT.^22, 23^ During metastasis, EMT can reduce the adhesion between cancer cells and enhance its ability to metastasis. EMT describes a biologic process that allows the epithelial cells to undergo multiple biochemical changes that enable them to lose their cell–cell basement membrane contacts and their structural polarity (epithelial-like phenotype) to assume the mesenchymal-like phenotype, accompanied with migratory capacity, invasiveness, elevated resistance to apoptosis and greatly increased production of extracellular matrix component.^24^ During EMT, downregulated E-cadherin and upregulated N-cadherin, termed as ‘cadherin switch,' is typically observed.^25^ In RCC, decreased E-cadherin expression is associated with metastasis.^22^ In this study, we show that HPIP overexpression modulates the invasion and metastasis of RCC cells, along with increased expression of N-cadherin and Vimentin and decreased expression of E-cadherin, suggesting that HPIP regulates RCC metastasis by activating the process of EMT. Again, CK1α is responsible for HPIP promotion of EMT.

The functions of CK1α in tumorigenesis are manifold, making it difficult to classify it as oncogene or tumor suppressor. In some types of cancer, CK1α seems to exhibit oncogenic features by promoting proliferation, genome instability and inhibition of apoptotic processes, such as ovarian cancer,^26^ acute myelocytic leukemia^27^ and so on, which are supported by the fact that CK1α is often overexpressed in those tumors and correlates with poor survival. Although in some other types of cancer, such as colorectal carcinoma, lymphomas and breast carcinomas,^28^ CK1α acts as a tumor suppressor which is evidenced by the fact that loss of heterozygosity of CK1α gene causes a highly invasive carcinoma and reduced CK1α expression was often observed in those tumors.^29^

## Materials and methods

### Immunohistochemistry

Renal cancer samples and adjacent noncancerous tissues were obtained from the China PLA 307 Hospital with the informed consent of patients. Before surgical therapy, none of the patients had received neoadjuvant chemotherapy, radiation therapy or immunotherapy. Diagnoses were based on pathological evidence. Study protocol was approved by the Ethics Committee of Chinese PLA 307 Hospital and all experimental methods were carried out in accordance with approved guidelines of Academy of Military Medical Sciences. The immunohistochemistry procedure was performed as described previously.^30^ Briefly, the antigens were retrieved by microwave treatment and incubated with rabbit anti-HPIP antibody (Proteintech, Rosemont, IL, USA) at a dilution of 1/100, or rabbit anti-CK1α antibody (Santa Cruz Biotechnology, Santa Cruz, CA, USA) at a dilution of 1/100. Bound primary antibodies were detected by the addition of biotinylated goat anti-rabbit secondary antibody and streptavidin-horseradish peroxidase (Zymed Laboratories, South San Francisco, CA, USA). The color was developed with 3,3′-diaminobenzidine. The samples were counterstained using hematoxylin. For negative controls, normal rabbit IgG (Santa Cruz Biotechnology) or phosphate-buffered saline was substituted for the primary antibody. All of immunohistochemistry staining was assessed by two pathologists blinded to the origination of the specimen. The widely accepted German semi-quantitative scoring system in considering the staining intensity and area extent was used:^30^ 0, no staining; 1, weak staining; 2, moderate staining; and 3, strong staining. In addition, the percentage of staining was given a score of 0 (<5%), 1 (5–25%), 2 (25–50%), 3 (51–75%) and 4 (>75%). Two scores mentioned above were multiplied as the final score. For HPIP, we defined 0 score as negative and 1–12 as positive. For CK1α, we defined 1–3 score as negative and 4–12 as positive.

### Plasmids, cell lines and reagents

Caki-1, 786-O and A498 cell lines were kind gifts from Professor Xu Zhang in the General Hospital of Chinese People's Liberation Army and tested for mycoplasma contamination. Stable cell lines overexpressing HPIP were established by lentiviral transduction using pCDH plasmid (System Biosciences). Stable HPIP or CK1α knockdown cell lines were established by cloning HPIP or CK1α shRNA fragment into the lentiviral vector pSIH-H1 (System Biosciences, Palo Alto, CA, USA). The sequence of HPIP shRNA has been described previously.^6^ The sequence of CK1α shRNA was AATCTCAGAAGGCCAGGCATC. Lentivirus was generated by transfection of the 293 T producer cell line with the lentiviral vector and packing vector mix (System Biosciences). Pooled clones were screened by immunoblot with anti-HPIP or anti-CK1α. Similar results were obtained with individual clones. Anti-HPIP was from Proteintech (12102-1-AP); anti-CK1α (#2655), anti-mTOR (#2972), anti-p-mTOR (S2448) (#2971), anti-S6K1 (#9202), anti-p-S6K1 (T389) (#9205) and anti-Vimentin (#3932) were from Cell Signaling Technology, Danvers, MA, USA; anti-E-cadherin (564186) and anti-N-cadherin (610920) were from BD Biosciences, Franklin Lakes, NJ, USA.

### Transient transfections

Cell lines were routinely cultured in recommended medium (DMEM for 293 T and Caki-1 cells, and RPMI 1640 for 786-O and A498 cells) supplemented with 10% fetal bovine serum at 37 °C in humidified atmosphere of 5% CO_2_ in air. Lipofectamine 2000 reagent and Lipofectamine RNAiMAX were used for transfections of plasmids and siRNAs, respectively, according to the manufacturer's guidelines (Invitrogen). siRNAs for CK1α were chemically synthesized (Invitrogen, Carlsbad, CA, USA).

### Yeast two-hybrid, GST pull-down and co-immunoprecipitation assays

Yeast two-hybrid assays were performed as previously described.^31^ For GST pull-down assay, GST fusion proteins were expressed and purified according to the manufacturers' instructions (Amersham Pharmacia and Qiagen, Buckinghamshire, CA, USA). Cell lysates were incubated with GST fusion protein bound to GST beads for 4 h at 4 °C. After washing, the precipitated components were analyzed by immunoblot. Co-immunoprecipitation (Co-IP) was performed as previously described.^31^

### Cell growth and colony formation assays

Anchorage-dependent cell growth was evaluated by the CCK-8 Kit (Dojindo Laboratories, Kumamoto, Japan) according to the manufacturer's instructions. For colony formation assays, transfected cells were seeded in six-well plates at 2000 cells per well. Two weeks later, the colonies were fixed with 4% paraformaldehyde and stained with a crystal violet solution for 30 min. The number of colonies containing at least 50 cells was counted. For anchorage-independent cell growth, a bottom layer of 0.7% low melting temperature agar and a top layer of 0.35% agar mixed with transfected cells were plated in six-well plates. Colonies with diameters >100 μm were counted after 3 weeks of growth.

### Cell migration and invasion assays

Wound healing assays were performed to assess cell migration. Briefly, transfected cells cultured in six-well plates as confluent monolayers were mechanically scratched using a 1-ml pipette tip to create the wound. Cells were washed with phosphate-buffered saline to remove the debris and were cultured for 16 h to allow wound healing. Cell invasion was determined with Matrigel (BD Biosciences) coated on the upper surface of the transwell chamber (Corning, New York, NY, USA). Twenty-four hours later, cells invaded through the Matrigel membrane were fixed with 4% paraformaldehyde and stained with crystal violet. The number of invaded cells was counted in five randomly selected microscopic fields and photographed.

### *In vivo* tumor growth and metastasis

All animal experiments were undertaken in accordance with the National Institute of Health Guide for the Care and Use of Laboratory Animals, with the approval of the Institutional Animal Care and Use Committee at Beijing Institute of Biotechnology. Caki-1 cells (2 × 10^7^) were injected into the hind limb of 6-week-old male nude mice (*n*=7), which was divided into two groups (*n*=7 based on minimal 30% decrease from 1 g tumors with 250 μg s.d., *α-*error of 0.05 and *β-*error of 0.8) using random number method with no blinding. Tumor size was measured at indicated times using calipers. Tumor volume was calculated according to the following formula: volume=(longest diameter × shortest diameter^2^)/2. For the metastasis model, 1 × 10^6^ Caki-1 cells stably infected with pSIH control or pSIH-HPIP shRNA were injected intravenously via the lateral tail vein of nude mice (*n*=6). All mice were maintained for about 50 days until analysis by the small-animal Positron emission tomography (PET) imaging system.

### Small-animal PET imaging

PET of tumor-bearing mice was performed using an animal PET scanner (Philips Corp., Amsterdam, Netherland).^32^ After intraperitoneally anesthetized by pentobarbital (100 mg/kg), mice were injected intravenously with 3.7 MBq (100 μCi) of ^18^F radio-labeled fluorodeoxyglucose (^18^F-FDG). Emission scans of 5-min duration were performed to obtain attenuation correction data in the prone position 1 h after injection, and 10-min delay scans were acquired at 2 h. For each mouse, radioactivity was calibrated against a known aliquot of the injected tracer and presented as percentage injected dose of tissue (% ID).

### Statistical analysis

All *in vitro* experiments were performed in triplicate and repeated three times. The difference of HPIP or CK1α expression between renal cancers and normal tissues was assessed by Mann–Whitney *U*-test. Estimation of disease-free survival and overall survival was performed using the Kaplan–Meier method, and differences between survival curves were examined with the log-rank test. Statistical significance in cell proliferation, apoptosis and invasion assays among constructs was determined by two-tailed Student's *t*-test. The SPSS 17.0 statistical software package was used to perform the statistical analyses. *P*<0.05 was considered statistically significant.

## Figures and Tables

**Table 1:** Clinical correlations of HPIP expression in renal carcinoma

*Variables*	*HPIP expression in tumor tissues (T)*			P*-value*
	*Low expression*	*High expression*	*Total*	
	N=*42 (35.3%)*	N=*77 (64.7%)*	N=*119 (100%)*	
Age (mean±s.d.) years	58.4±6.92	59.2±8.76	58.7±7.84	0.866
				
*Tumor size (cm)*				
<5	30 (51.7%)	28 (48.3%)	58	2.550 × 10^−4a^
⩾5	12 (19.7%)	49 (80.3%)	61	
				
*Differentiation*				
High	23 (33.8%)	45 (66.2%)	68	0.804
Moderate	16 (39.0%)	25 (61.0%)	41	
Poor	3 (30.0%)	7 (70.0%)	10	
				
*Histology*				
Clear-cell carcinoma	31 (32.6%)	64 (67.4%)	95	0.401
Adenocarcinoma	8 (50.0%)	8 (50.0%)	16	
Other	3 (37.5%)	5 (62.5%)	8	
				
*Fuhrrman grades*				
I/II	25 (58.1%)	18 (41.9%)	43	8.759 × 10^−5a^
III/IV	17 (22.4%)	59 (77.6%)	76	
				
*Preoperative metastasis^b^*				
Absent	36 (43.4%)	47 (56.6%)	83	0.005^a^
Present	6 (16.7%)	30 (83.3%)	36	

*P*-values of age were calculated by Student's *t*-test and others by Pearson's Chi-square test.

a Statistically significant.

b Preoperative metastasis indicates preoperative local lymphatic metastasis and distant metastasis.

## References

[bib1] Capitanio U, Montorsi F. Renal cancer. Lancet 2015; 387: 894–906. 26318520 10.1016/S0140-6736(15)00046-X

[bib2] Bugide S, David D, Nair A, Kannan N, Samanthapudi VS, Prabhakar J et al. Hematopoietic PBX-interacting protein (HPIP) is overexpressed in breast infiltrative ductal carcinoma and regulates cell adhesion and migration through modulation of focal adhesion dynamics. Oncogene 2015; 34: 4601–4612. 25486428 10.1038/onc.2014.389

[bib3] Wang X, Yang Z, Zhang H, Ding L, Li X, Zhu C et al. The estrogen receptor-interacting protein HPIP increases estrogen-responsive gene expression through activation of MAPK and AKT. Biochim Biophys Acta 2008; 1783: 1220–1228. 18302941 10.1016/j.bbamcr.2008.01.026

[bib4] van Vuurden DG, Aronica E, Hulleman E, Wedekind LE, Biesmans D, Malekzadeh A et al. Pre-B-cell leukemia homeobox interacting protein 1 is overexpressed in astrocytoma and promotes tumor cell growth and migration. Neuro Oncol 2014; 16: 946–959. 24470547 10.1093/neuonc/not308 PMC4057136

[bib5] Xu X, Jiang C, Wang S, Tai Y, Wang T, Kang L et al. HPIP is upregulated in liver cancer and promotes hepatoma cell proliferation via activation of G2/M transition. IUBMB Life 2013; 65: 873–882. 24038948 10.1002/iub.1202

[bib6] Xu X, Fan Z, Kang L, Han J, Jiang C, Zheng X et al. Hepatitis B virus X protein represses miRNA-148a to enhance tumorigenesis. J Clin Invest 2013; 123: 630–645. 23321675 10.1172/JCI64265 PMC3561812

[bib7] Okada S, Irié T, Tanaka J, Yasuhara R, Yamamoto G, Isobe T et al. Potential role of hematopoietic pre-B-cell leukemia transcription factor-interacting protein in oral carcinogenesis. J Oral Pathol Med 2015; 44: 115–125. 25060351 10.1111/jop.12210

[bib8] Feng Y, Xu X, Zhang Y, Ding J, Wang Y, Zhang X et al. HPIP is upregulated in colorectal cancer and regulates colorectal cancer cell proliferation, apoptosis and invasion. Sci Rep 2015; 5: 9429. 25800793 10.1038/srep09429 PMC4371107

[bib9] Schittek B, Sinnberg T. Biological functions of casein kinase 1 isoforms and putative roles in tumorigenesis. Mol Cancer 2014; 13: 231. 25306547 10.1186/1476-4598-13-231 PMC4201705

[bib10] Lamouille S, Derynck R. Cell size and invasion in TGF-beta-induced epithelial to mesenchymal transition is regulated by activation of the mTOR pathway. J Cell Biol 2007; 178: 437–451. 17646396 10.1083/jcb.200611146 PMC2064840

[bib11] Perl A. mTOR activation is a biomarker and a central pathway to autoimmune disorders, cancer, obesity, and aging. Ann N Y Acad Sci 2015; 1346: 33–44. 25907074 10.1111/nyas.12756 PMC4480196

[bib12] Lee JJ, Loh K, Yap YS. PI3K/Akt/mTOR inhibitors in breast cancer. Cancer Biol Med 2015; 12: 342–354. 26779371 10.7497/j.issn.2095-3941.2015.0089 PMC4706528

[bib13] Duan S, Skaar JR, Kuchay S, Toschi A, Kanarek N, Ben-Neriah Y et al. mTOR generates an auto-amplification loop by triggering the βTrCP-and CK1α-dependent degradation of DEPTOR. Mol Cell 2011; 44: 317–324. 22017877 10.1016/j.molcel.2011.09.005 PMC3212871

[bib14] Knippschild U, Krüger M, Richter J, Xu P, García-Reyes B, Peifer C et al. The CK1 family: contribution to cellular stress response and its role in carcinogenesis. Front Oncol 2014; 4: 96. 24904820 10.3389/fonc.2014.00096 PMC4032983

[bib15] Elyada E, Pribluda A, Goldstein RE, Morgenstern Y, Brachya G, Cojocaru G et al. CKIα ablation highlights a critical role for p53 in invasiveness control. Nature 2011; 470: 409–413. 21331045 10.1038/nature09673

[bib16] Brockschmidt C, Hirner H, Huber N, Eismann T, Hillenbrand A, Giamas GC et al. Anti-apoptotic and growth-stimulatory functions of CK1 delta and epsilon in ductal adenocarcinoma of the pancreas are inhibited by IC261 *in vitro* and *in vivo*. Gut 2008; 57: 799–806. 18203806 10.1136/gut.2007.123695

[bib17] Knippschild U, Wolff S, Giamas G, Brockschmidt C, Wittau M, Würl PU et al. The role of the casein kinase 1 (CK1) family in different signaling pathways linked to cancer development. Onkologie 2005; 28: 508–514. 16186692 10.1159/000087137

[bib18] Foldynová-Trantírková S, Sekyrová P, Tmejová K, Brumovská E, Bernatík O, Blankenfeldt W et al. Breast cancer-specific mutations in CK1 epsilon inhibit Wnt/beta-catenin and activate the Wnt/Rac1/JNK and NFAT pathways to decrease cell adhesion and promote cell migration. Breast Cancer Res 2010; 12: R30. 20507565 10.1186/bcr2581 PMC2917022

[bib19] Cho D. Novel targeting of phosphatidylinositol 3-kinase and mammalian target of rapamycin in renal cell carcinoma. Cancer J 2013; 19: 311–315. 23867512 10.1097/PPO.0b013e31829d5cea PMC3934425

[bib20] Su D, Stamatakis L, Singer EA, Srinivasan R. Renal cell carcinoma: molecular biology and targeted therapy. Curr Opin Oncol. 2014; 26: 321–327. 24675233 10.1097/CCO.0000000000000069 PMC4232438

[bib21] Amato R, Stepankiw M. Evaluation of everolimus in renal cell cancer. Expert Opin Pharmacother. 2013; 14: 1229–1240. 23578333 10.1517/14656566.2013.791677

[bib22] Mikami S, Oya M, Mizuno R, Kosaka T, Katsube K, Okada Y. Invasion and metastasis of renal cell carcinoma. Med Mol Morphol. 2014; 47: 63–67. 24213520 10.1007/s00795-013-0064-6

[bib23] De Chiara L, Crean J. Emerging transcriptional mechanisms in the regulation of epithelial to mesenchymal transition and cellular plasticity in the kidney. J Clin Med 2016; 5: 6. 10.3390/jcm5010006 PMC4730131 26771648

[bib24] Smith BN, Bhowmick NA. Role of EMT in metastasis and therapy resistance. J Clin Med 2016; 5: 17. 10.3390/jcm5020017 PMC4773773 26828526

[bib25] Smith AP, Verrecchia A, Fagà G, Doni M, Perna D, Martinato F et al. A positive role for Myc in TGFβ-induced Snail transcription and epithelial-to-mesenchymal transition. Oncogene 2009; 28: 422–430. 18978814 10.1038/onc.2008.395

[bib26] Rodriguez N, Yang J, Hasselblatt K, Liu S, Zhou Y, Rauh-Hain JA et al. Casein kinase I epsilon interacts with mitochondrial proteins for the growth and survival of human ovarian cancer cells. EMBO Mol Med 2012; 4: 952–6310. 22707389 10.1002/emmm.201101094 PMC3491827

[bib27] Jaras M, Miller PG, Chu LP, Puram RV, Fink EC, Schneider RK et al. Csnk1a1 inhibition has p53-dependent therapeutic efficacy in acute myeloid leukemia. J Exp Med 2014; 211: 605–1210. 24616378 10.1084/jem.20131033 PMC3978274

[bib28] Sinnberg T, Menzel M, Kaesler S, Biedermann T, Sauer B, Nahnsen S et al. Suppression of casein kinase 1 alpha in melanoma cells induces a switch in beta-catenin signaling to promote metastasis. Cancer Res 2010; 70: 6999–7009. 20699366 10.1158/0008-5472.CAN-10-0645

[bib29] Elyada E, Pribluda A, Goldstein RE, Morgenstern Y, Brachya G, Cojocaru G et al. CKI alpha ablation highlights a critical role for p53 in invasiveness control. Nature 2011; 470: 409–1310. 21331045 10.1038/nature09673

[bib30] Pan X, Zhou T, Tai YH, Wang C, Zhao J, Cao Y et al. Elevated expression of CUEDC2 protein confers endocrine resistance in breast cancer. Nat Med 2011; 17: 708–714. 21572428 10.1038/nm.2369

[bib31] Zhang H, Xie X, Zhu X, Zhu J, Hao C, Lu Q et al. Stimulatory cross-talk between NFAT3 and estrogen receptor in breast cancer cells. J Biol Chem 2005; 280: 43188–43197. 16219765 10.1074/jbc.M506598200

[bib32] Kang L, Fan Z, Sun H, Feng Y, Ma C, Yan P et al. Improved synthesis and biological evaluation of Tc-99 m radiolabeled AMO for miRNA imaging in tumor xenografts. J Labelled Comp Radiopharm 2015; 58: 461–468. 26503645 10.1002/jlcr.3351

